# Perceived Health and Nutrition Concerns as Predictors of Dietary Patterns among Polish Females Aged 13–21 Years (GEBaHealth Project)

**DOI:** 10.3390/nu9060613

**Published:** 2017-06-16

**Authors:** Marzena Jezewska-Zychowicz, Lidia Wadolowska, Joanna Kowalkowska, Marta Lonnie, Jolanta Czarnocinska, Ewa Babicz-Zielinska

**Affiliations:** 1 Department of Organization and Consumption Economics, Warsaw University of Life Sciences, Nowoursynowska 159 C, 02-776 Warsaw, Poland; marzena_jezewska_zychowicz@sggw.pl; 2 Department of Human Nutrition, University of Warmia and Mazury in Olsztyn, Sloneczna 45F, 10-718 Olsztyn, Poland; lidia.wadolowska@uwm.edu.pl (L.W.), joanna.kowalkowska@uwm.edu.pl (J.K.); 3 Department of Human Nutrition and Hygiene, Poznan University of Life Sciences, Wojska Polskiego 28, 60-637 Poznan, Poland; jolanta.czarnocinska@up.poznan.pl; 4 Faculty of Physiotherapy and Health Sciences, Gdansk Management College, Pelplinska 7, 80-335 Gdansk, Poland; zielarz.47@wp.pl

**Keywords:** dietary patterns, females, health concerns, Health Concerns Scale, nutrition concerns, PCA

## Abstract

Health-related concerns can often be factors influencing health-related behaviours. It remains unclear whether a high level of concerns is associated with pro-healthy or unhealthy dietary behaviours and whether any associations between nutrition-related concerns and dietary behaviours exist in a population of girls and young women. The aim of the study was to investigate the associations between perceived health and nutrition concerns and dietary patterns in a representative sample of Polish young females. Data was collected in 2012 through a cross-sectional quantitative survey within the GEBaHealth (Girls Eating Behaviours and Health) project in a group of 1107 Polish girls aged 13–21 years old. Dietary patterns were identified by Principal Component Analysis (PCA) based on dietary data collected with Food Frequency Questionnaires (FFQs). Nutrition and health concerns were assessed separately by two indices: Health Concern Index (HCI) and Nutrition Concern Index (NCI); both based on the Health Concern Scale (HCS). The associations between perceived health and nutrition concerns and each dietary pattern were investigated using logistic regression analysis. Displaying a higher level of health concerns increased the chances of adherence to the upper tertile of ‘Fruit & vegetables’ pattern (adjusted odds ratio [adj. ORs]: 1.46, 95% Confidence Interval [95% CI]: 1.02–2.10). Displaying a lower level of health concerns increased the chances of the adherence to the upper tertiles of ‘Traditional Polish’, ‘Dairy & fats’, ‘Fruit and vegetables’ and ‘Fast food & sweets’ patterns (adj. ORs: 1.87, 95% CI: 1.31–2.67; 1.66, 95% CI: 1.18–2.34; 1.57, 95% CI: 1.11–2.22; 1.52, 95% CI: 1.08–2.13; respectively). No significant associations were found between levels of nutrition concerns and dietary patterns in the adjusted model. We found associations between self-perceived health concerns and dietary patterns in our study sample, suggesting health concerns can be an important predictor of dietary behaviours in girls and young women. To increase the effectiveness of healthy eating, an emphasis should be laid on health, reinforced with awareness of nutrition, when advising on food-related decisions.

## 1. Introduction

The public interest in health is increasing, but the rising prevalence of diet-related non-communicable diseases including obesity can still be observed. Interventions aimed at treating obesity have demonstrated only modest effects over time [1] particularly in high-risk groups [2]. While interventions to reduce sedentary behaviors, particularly television viewing, have been more successful [3], studies indicate that there is a need for gaining a better understanding of healthy diet predictors, especially in at-risk populations such as adolescents, to increase the prevalence of positive health behaviors [4,5,6].

An unhealthy diet, characterized by high sugar and fat intake and low intake of fruit and vegetables is one of the leading causes of non-communicable disease globally [7] and is strongly associated with early mortality [8,9]. Poor dietary patterns are a key factor in the obesity epidemic [10], also among young people [11]. The transition from adolescence to young adulthood has been identified as a critical time during which some less healthful behavioral patterns are developed [12]. Young people aged 18–24 years may underestimate the effect of their current actions on their long-term health status and exhibit vulnerable attitudes to potential risk factors [13]. Moreover, the results of some studies indicate that young girls tend to use various quantitative and qualitative dietary restrictions [14] that may lead to unhealthy dietary habits and eating disorders.

The self-perceived importance of health and nutrition can have a vital influence on one’s behaviors. Different terminology has been used to evaluate its impact on eating habits, e.g., ‘health interests’ [15] or ‘health concern’ [16,17]. Regardless of the terminology employed, these conceptualizations reflect either the importance or concerns about health, nutrition, and food. A great deal of research has been focused mostly on understanding consumer perception of food risks, and the number of such investigations has increased in the later decades due to various reasons. For instance, highly processed food has been intensively introduced to the market, but also food has been involved in many food crises [18,19]. Risks and concerns connected to one’s own lifestyle are often downplayed compared to risks connected to the society [18]. Most people think that their nutritional behaviors are under their control, so perceived health and nutrition risks may not have an impact on their dietary behaviors. It seems, however, that within the population of females of pre- and reproductive age the perceived concern about diet and health may be of a higher importance comparing to others. Firstly, dietary habits established in youth may not only track to the women’s adult life [20] but be also passed down to their children [21]. Secondly, there is still a need for developing effective prevention programs for improving the diet of young females (and subsequently their children) to improve their nutritional status and subsequently to reduce the rates of obesity or malnutrition.

In our study, we hypothesized that females who had a high level of concerns about health or nutrition may present specific (either healthy or unhealthy) dietary behaviors. The rationale for this hypothesis can be twofold. First, a high level of concerns may be an important motive in establishing and maintaining healthy dietary behaviors. And second, a high level of concerns might be associated with e.g., decline in health caused by following an unhealthy diet. To date, limited evidence has been reported describing the associations between perceived concerns about health and nutrition, and dietary patterns, especially among young females. Thus, the objective of our study was to investigate the associations between perceived health and nutrition concerns and previously derived dietary patterns. 

## 2. Materials and Methods 

### 2.1. Study Sample 

Data was collected in 2012 through a cross-sectional quantitative survey within the GEBaHealth (Girls Eating Behaviors and Health) project [22]. According to the study design, recruitment and data collection were made by the Public Opinion Research Center (CBOS, Warsaw, Poland) [23]. A group of 2104 Polish females aged 13–21 years old was recruited from Universal Electronic System of Population Register (PESEL) [24]. The computer-assisted personal interviewing (CAPI) technique was used to collect all data. Due to refusals, absence, disability, and other reasons, 47.4% of initially contacted respondents (*n* = 997) did not take part in the study. Details regarding final sample (*n* = 1107), recruitment protocol and methodology have been previously reported [22].

### 2.2. Ethical Approval

The Bioethics Committee of the Faculty of Medical sciences, University of Warmia and Mazury in Olsztyn, approved the protocol of the GEBaHealth study on the 17th of June 2010, Resolution No. 20/2010. Informed consent was provided by adult participants (18+ years) and/or under-aged participants’ parents or legal guardians (<18 years).

### 2.3. Perceived Concerns about Health and Nutrition

To evaluate the level of concerns about health and nutrition two indices were created using the original Health Concern Scale [25]. The Health Concerns Index (HCI) included three statements: (1) I am concerned about gaining weight; (2) I am concerned about the risk of high blood pressure and (3) I am concerned about the risk of coronary heart disease. The Nutrition Concern Index (NCI) included seven statements: (1) I am concerned about getting many calories; (2) I am concerned about getting a lot of fat in my food; (3) I am concerned about getting a lot of cholesterol in my food; (4) I am concerned about getting a lot of sugar in my food; (5) I am not concerned about getting a lot of salt in my food (re-coded—accordingly to the original reference [25]); (6) I am concerned about getting sufficient energy in my food; and (7) I am concerned about food additives in my food. Participants estimated their own concerns on a 7-point Likert scale starting from ‘definitely not’ (0 points assigned) through ‘neither not nor yes’ (3 points) to ‘definitely yes’(6 points). Then, the sum of the ratings for each index was calculated. The range for HCI was 0–18 points, and for NCI 0–42 points. Because of the skewed distribution of HCI and NCI participants were divided into three groups based on tertile distribution (supplementary Table S1).

### 2.4. Dietary Patterns

Data was collected using three food-frequency questionnaires: Block Screening Questionnaire for Fruit/Vegetable/Fibre Intake (BSQFVF), Block Screening Questionnaire for Fat Intake (BSQF) and Food Intake Variety Questionnaire (FIVeQ). From these questionnaires, data regarding 30 food items were collected. Using Principal Component Analysis (PCA) four dietary patterns were derived: ‘Traditional Polish’ (comprising of white bread, potatoes, red meat, margarine/butter, and fried chicken), ‘Fruit and vegetables’ (comprising of salads, fruit, vegetables, and beans), ‘Fast foods and sweets’ (comprising of fries, chips, burgers, ice-cream, pastries/cakes, salad dressings, sweets, and snacks) and ‘Dairy and fats’ (comprising of cheese, whole milk, margarine/butter, cereals and potatoes, and dairy products) (supplementary Table S2). Dietary data collection and detailed methodology regarding dietary patterns identification were previously described in details [22].

### 2.5. Confounding Variables

Socioeconomic status was assessed by socioeconomic status index (SESI) [26] based on questions regarding mother’s education, father’s education, economic status and description of household. Body Mass Index (BMI) was calculated using self-reported weight and height and categorised accordingly to the International Obesity Task Force (IOTF) standards [27]. Details regarding data collection and cut-offs for confounding variables such as age, socioeconomic status and BMI were previously described [22,26].

### 2.6. Statistical Analyses 

Descriptive statistics including frequency distributions and cross-tabulations were carried out. The Principal Component Analysis (PCA), varimax rotation, was applied to derive dietary patterns. Girls in each of the dietary patterns were divided into three groups based on tertile distribution: bottom, middle and upper tertile; girls in upper tertile had the strongest adherence to the pattern [22]. A simple logistic regression analysis was applied to verify the associations between the tertiles of both concerns indices (HCI and NCI) and tertiles of each dietary pattern. For each analysis, two categories of independent variables and two categories of dependent variables were included. Results were reported as odds ratio (OR) with 95% confidence intervals (95% CI). The reference groups were participants representing middle tertile of health or nutrition concerns (OR = 1.00) and bottom tertiles of dietary patterns (OR = 1.00). The significance of ORs was assessed by Wald’s statistics. Two models were created: (i) crude and (ii) adjusted for age and SES index (as continuous variables) and for BMI (as a categorical variable, due to the heterogeneity of the sample which included adolescents and adults).

Variables normality was checked by Kolmogorov–Smirnov test. Continuous data are presented as means. The differences between groups were verified by the significance level of Kruskal–Wallis test. Analyses were conducted with sample weights to correct non-response data (in respect to sociodemographic characteristics available in the PESEL database) during the sample collection. Sample weights were calculated by statisticians from CBOS research agency, using mathematical modelling. For all tests, *p*-value < 0.05 was considered as significant. The statistical analysis was carried out using STATISTICA software (version 10.0 PL; StatSoft Inc., Tulsa, OK, USA; StatSoft Polska, Krakow, Poland).

## 3. Results

Mean value of HCI was 6.9 points and NCI was 17.1 points, both means were located in the middle tertiles and within ranges labelled as neutral concerns (Table 1, supplementary Table S1). Both HCI and NCI were significantly differentiated across tertiles—the HCI by all three statements and the NCI by six from seven statements, except statement referring to the lack of attention for salt in food. 

Significant differences across tertiles of HCI were observed in terms of specific age groups, BMI categories and each dietary pattern (Table 2). Significant differences across tertiles of NCI were observed in terms of age, BMI categories and SES index. No significant differences between tertiles of NCI and tertiles of each dietary pattern were revealed. Obese or overweight females in comparison to normal weight demonstrated significantly higher HCI (9.7 and 9.4 vs. 6.8 points, respectively) as well as NCI (20.2 and 21.3 vs 16.9 points, respectively) (supplementary Table S3).

Table 3 reports the results from crude and adjusted (for age, SES and BMI) logistic regression models showing the associations between tertiles of HCI and NCI and tertiles of four dietary patterns.

The results from the crude model have demonstrated that girls who displayed a high level of health concerns (the upper tertile of HCI) were more likely to fall in the upper tertile of ‘Fruit and vegetables’ pattern and less likely to fall in the middle tertile of ‘Dairy and fats’ pattern (crude ORs: 1.61, 1.15–2.24 and 0.68, 0.49–0.95, respectively). In contrast, girls who displayed a low level of health concerns (bottom tertile of HCI) were more likely to fall in the middle and upper tertiles of ‘Traditional Polish’ pattern (crude ORs: 1.46, 1.05–2.05 and 1.90, 1.37–2.64, respectively), and upper tertiles of ‘Fast foods and sweets’, ‘Dairy and fats’ and ‘Fruit and vegetables’ patterns (crude ORs: 1.66, 1.20–2.30; 1.63, 1.17–2.26; 1.42, 1.03–1.97, respectively). Associations between the tertiles of NCI and dietary patterns in the crude model were observed in regards to two patterns; girls in a bottom tertile of NCI were more likely to fall in the upper tertile of ‘Fast food and sweets’ pattern and less likely to fall in the upper tertile of ‘Fruit and vegetables’ pattern (crude ORs: 1.43, 1.03–1.98 and 0.69, 0.49–0.96, respectively).

Consistently, the results from the adjusted model have shown that girls who displayed a high level of health concerns (the upper tertile of HCI) were more likely to fall in the upper tertile of ‘Fruit and vegetables’ and less likely to fall in the middle tertile of ‘Dairy and fats’ patterns (adjusted ORs: 1.46, 1.02–2.10 and 0.69, 0.48–0.98) and girls who perceived the level of health concerns as low (the bottom tertile of HCI) had increased chances of falling in the upper tertiles of ‘Traditional Polish’, ‘Dairy and fats’, ‘Fruit and vegetables’ and ‘Fast foods and sweets’ patterns (adj. ORs: 1.87, 95% CI 1.31–267; 1.66, 1.18–2.34; 1.57, 1.11–2.22; 1.52, 1.08–2.13, respectively). In the adjusted model, no significant associations were found between the tertiles of NCI and dietary patterns.

## 4. Discussion

Results of this study verified our hypothesis, demonstrating that females who displayed a high level of health concerns presented healthier dietary behaviours than females who were not overly concerned about their health. Furthermore, a low level of health concerns was linked with undesirable dietary behaviours. In terms of nutrition concerns no significant associations with dietary patterns were found in the adjusted model, which indicates that health concerns were stronger predictors of dietary behaviours than nutrition concerns.

In recent years, a significant increase in concerns about health and wellness and a corresponding growth in consumer demand for healthy food products have been observed [28]. Previous studies suggested that consumers’ health concerns are closely related to food consumption [16,29,30]. This relation was found in our study among young females. Those who were concerned about health presented pro-healthy dietary behaviours, which was reflected by a greater chance of frequent fruits, vegetables, and beans intake. The relationship between high concerns about health and greater intake of fruit and vegetables is easier to explain in the light of the results of previous studies. It has been demonstrated, that young people tend to consider fruits and vegetables as healthy foods, and therefore the increase in the consumption is often observed among those who wish to improve or maintain their physical and cognitive performance [31]. In contrast, a low level of health concerns favoured the adherence to all of the dietary patterns, including pro-healthy ‘Fruit and vegetables’ pattern as well as rather unhealthy and unhealthy dietary patterns characterised by frequent consumption of either: stodgy traditional Polish foods, full-fat dairy and fats or fast foods and sweets. The association between low level of health concerns and unhealthy dietary pattern was anticipated and is in line with previous studies. For instance, Hartmann et al. [32] found that women who were in the least health-conscious group, adhered to the ‘Unhealthy pattern’, described by the two characteristics: frequent consumption of unhealthy foods and a low consumption of fruit and vegetables. Our finding adds to the existing evidence, indicating that the association between low level of health concerns and unhealthy dietary pattern may have already been established at younger age.

Moreover, the surprising finding that did not support previous research, was that a low level of health concerns was increasing the chances of more frequent fruit and vegetables consumption. The potential explanation for this phenomenon may not be straightforward. It could be, that in this specific population, the increased frequency of fruit and vegetables consumption (that was coexisting with unhealthy dietary behaviours) may not have been a result of health-consciousness, but could have been of an environmental nature. It has been shown, that parental intake of fruit and vegetables and so-called ‘parental modelling’ is associated with fruit and vegetables intake in adolescents [33]. Due to the young age of study participants, the majority of them still lived in their family home. Hence, family environment could have been a potential confounding factor, affecting frequency of fruit and vegetable intake. The alternative explanation could be, that the included indicators of perceived concern about health included in our study (such as gaining weight, risk of high blood pressure, and risk of coronary heart disease) could not be related by the respondents to the consumption of fruit and vegetables. Thus, the assumptions of many models of preventive health behavior were not reflected; for instance, the Health Belief Model which states that the more serious a person perceives personal risk from a behavior, the more receptive is to any cues concerning the behavior, which potentially derives appropriate action [34].

Results of our study have shown that the level of nutrition concern did not significantly differentiate participant’s’ adherence to the dietary patterns. However, in the crude model, girls with low concerns about nutrition were more likely to present unhealthy behaviors, i.e., had lower chances of frequent fruit and vegetables consumption and higher chances of frequent fast food and sweets consumption. The fact that concerns about nutrition differentiated adherence to separate dietary patterns only to a small extent may result from its origin. Low nutrition concern may stem from a low awareness among young people about the nutritional recommendations and the relation between diet and health. Research shows that students do not have adequate knowledge of age- and sex-specific nutrition recommendations [16,35]. The lack of sufficient knowledge about adequate nutrition combined with the occurrence of unhealthy eating behaviors can coexist with a lack of ability to assess one’s behavior and result in a low level of concerns about nutrition. 

In girls and young women under study, health concerns determined the prevalence of dietary patterns more than nutrition concerns. This is in line with the results of Hoefkens et al. [36] indicating that adult Europeans underestimate the importance of eating habits for personal health. Other researches point out that individuals with concerns of developing diseases would place more importance on health, price, natural content and ethical issues in making food-related decisions, which may result in more positive attitudes toward healthy eating [16].

An interesting finding, unrelated to the aim of this study, was that apart from the dietary patterns, BMI significantly differentiated perceived concerns about health and nutrition in girls and young women. The significantly higher health and nutrition concerns were observed for those who were overweight or obese. The study by Finkelstein et al. [37] reported associations between the excess weight and greater self-perceived risk of diseases such as cancer, heart diseases, and stroke. Perhaps, experienced health problems and dietary restrictions implemented by people with excess body weight contribute to raising awareness of the problem, developing concern for health and evaluating their diet [4,34]. The relation between overweight or obesity and the perceived concerns about health or nutrition is worth further studying. In our study we used BMI as one of the confounding variables, to minimize this effect.

### Strengths & Limitations

The current study had a number of strengths. First, it benefits from a large, nationally representative sample of 1107 girls and young women. Secondly, to our knowledge this is the first study that analyzed dietary patterns in a context of two separate categories of self-perceived concerns: health- and nutrition-related. We believe that this distinction brought a new perspective, indicating that nutrition concerns are not strong enough predictors of dietary behavior among young females. Lastly, although our results could be only extrapolated to a Polish population, the observations described could be of potential use in designing interventions in countries, that similar to Poland, do not have a long history of nutritional education in public schools. 

Study limitations also need to be taken into account. We have used FFQs to collect dietary data and it has been previously shown that self-reported data is often biased by overestimation of pre-healthy foods and underestimation of foods considered us unhealthy [38]. However, the FFQ method is still the most commonly used tool for long-term dietary intake assessment, and this approach is more valuable when investigating the psychological factors affecting dietary behaviors. Nevertheless, to minimize the impact of bias, we have chosen validated questionnaires and adjusted the analyses for potential confounders. Another limitation was that the assessment of health and nutrition concerns was based on the limited number of questions. Future researchers might seek to determine the effects of various manifestations of health concern. Besides the concern of developing diseases, consuming too many calories and consuming inadequate amounts of food, perhaps other types of health concerns can also predict dietary patterns.

## 5. Conclusions

We found significant associations between health concerns and dietary patterns in Polish young females, which suggests their importance in the establishment of eating habits. Girls and young women with higher level of concerns about health presented healthier dietary patterns than those with a lower level of health concerns. An interesting finding was that high adherence to ‘Fruit and vegetables’ pattern was found not only among females with a higher level of health concerns, but also among those less concerned about health, which indicate that in the latter group the intake was not necessarily determined by health-consciousness, but could have been based on dietary habits established at home. To increase the effectiveness of healthy eating, an emphasis should be laid on health, reinforced with awareness of nutrition, when advising on food-related decisions.

## Figures and Tables

**Table 1:** Perceived health and nutrition concerns by tertiles (mean (SD), in points).

Indices and Its Statements ^a^	Total Sample *n* = 1107	Bottom Tertile *n* = 355/339	Middle Tertile *n* = 424/417	Upper Tertile *n* = 328/351	*p*-Value
**Perceived health concerns**					
Health Concern Index ^b^	6.9 (3.7)	3.0 (0.7)	6.6 (0.9)	11.6 (2.3)	<0.0001
I am concerned about gaining weight	3.1 (1.9)	1.0 (0.5)	3.6 (1.5)	4.6 (1.2)	<0.0001
I am concerned about the risk of high blood pressure	2.0 (1.4)	1.0 (0.3)	1.5 (0.7)	3.7 (1.3)	<0.0001
I am concerned about the risk of coronary heart disease	1.8 (1.3)	1.0 (0.3)	1.4 (0.8)	3.3 (1.4)	<0.0001
**Perceived nutrition concerns**					
Nutrition Concern Index ^c^	17.1 (6.6)	10.0 (1.7)	16.1 (1.9)	25.1 (4.2)	<0.0001
I am not concerned about getting a lot of salt in my food (re-coded)	3.7 (1.5)	3.4 (1.8)	3.8 (1.4)	3.7 (1.4)	0.1617
I am concerned about food additives in my food	2.6 (1.5)	1.4 (0.9)	2.7 (1.4)	3.5 (1.4)	<0.0001
I am concerned about getting a lot of sugar in my food	2.4 (1.6)	1.1 (0.7)	2.1 (1.2)	4.2 (1.2)	<0.0001
I am concerned about getting a lot of fat in my food	2.3 (1.6)	1.0 (0.4)	2.0 (1.3)	4.0 (1.3)	<0.0001
I am concerned about getting sufficient energy in my food	2.2 (1.5)	1.1 (0.5)	2.2 (1.3)	3.2 (1.5)	<0.0001
I am concerned about getting a lot of cholesterol in my food	2.1 (1.5)	1.0 (0.3)	1.7 (1.0)	3.6 (1.3)	<0.0001
I am concerned about getting many calories	1.9 (1.3)	1.0 (0.5)	1.7 (1.0)	2.9 (1.5)	<0.0001

SD-standard deviation. All data adjusted for sample weights. ^a^ Sorted by means of statements. ^b^ Index range: 0–18 points. ^c^ Index range: 0–42 points, both indices were calculated as a sum of points assigned to each statements based on 7-point Likert scale starting from ‘definitely not’ (0 point) through ‘neither not nor yes’ (3 points) to ‘definitely yes’ (6 points). *n*-number, in the brackets participants’ number for tertiles of Health/ Nutrition Concern Index is given. *p*-value: significance level of Kruskal–Wallis test.

**Table 2:** Sample distribution within socioeconomic status, body weight status and dietary patterns by perceived health and nutrition concerns (%).

Variable		Total *n* = 1107	Health Concern Index				Nutrition Concern Index			
			Bottom Tertile *n* = 355	Middle Tertile *n* = 424	Upper Tertile *n* = 328	*p*-Value	Bottom Tertile *n* = 339	Middle Tertile *n* = 417	Upper Tertile *n* = 351	*p*-Value
Total sample		100.0	32.1	38.3	29.6		30.6	37.6	31.7	
Age (years)	Mean (SD)	17.3 (2.6)	17.3 (2.6)	17.0 (2.6)	17.6 (2.5)	0.0118	17.3 (2.6)	17.2 (2.6)	17.4 (2.5)	0.4280
Age (categories)	13–15 years	29.5	31.4	33.8	21.8	0.0044	26.5	34.1	26.8	0.0383
	16–18 years	33.1	30.4	31.2	38.5		37.8	28.8	33.7	
	19–21 years	37.4	38.2	35.0	39.7		35.7	37.1	39.5	
Residence	Rural area	47.1	43.4	49.1	48.4	0.2003	48.4	44.6	48.8	0.5988
	Town	31.4	36.0	30.1	28.2		32.3	32.3	29.5	
	City	21.5	20.6	20.8	23.4		19.3	23.1	21.7	
SES Index ^a^	Low	36.2	37.5	33.5	38.2	0.4263	39.3	32.7	37.3	0.0131
	Medium	30.6	31.5	30.0	30.6		31.5	27.7	33.3	
	High	33.2	31.0	36.5	31.2		29.2	39.6	29.4	
BMI category ^b^	Underweight	10.2	19.2	8.5	2.6	< 0.0001	15.1	12.0	3.1	< 0.0001
	Normal weight	77.7	75.3	83.8	72.5		79.8	78.6	74.8	
	Overweight	10.5	4.4	7.4	21.1		4.5	8.2	19.0	
	Obese	1.6	1.1	0.3	3.8		0.6	1.2	3.1	
Dietary patterns										
‘Traditional Polish’	Bottom tertile	33.1	24.2	36.4	38.5	< 0.0001	29.9	34.5	34.6	0.1787
	Middle tertile	32.9	33.5	32.4	32.9		31.1	32.7	34.9	
	Upper tertile	34.0	42.3	31.2	28.6		39.0	32.8	30.5	
‘Fruit and vegetables’	Bottom tertile	32.9	32.2	37.1	28.2	0.0201	37.6	30.7	31.0	0.0771
	Middle tertile	33.2	31.4	34.5	33.3		34.0	33.9	31.5	
	Upper tertile	33.9	36.4	28.4	38.5		28.4	35.4	37.5	
‘Fast foods and sweets’	Bottom tertile	33.0	28.1	34.2	36.7	0.0081	29.7	33.3	35.8	0.0737
	Middle tertile	33.0	30.7	35.7	32.0		30.4	35.5	32.6	
	Upper tertile	34.0	41.2	30.1	31.3		39.9	31.2	31.6	
‘Dairy and fats’	Bottom tertile	33.1	27.5	33.8	38.1	0.0017	31.4	33.3	34.4	0.8899
	Middle tertile	32.9	32.1	37.1	28.6		34.9	32.5	31.6	
	Upper tertile	34.0	40.4	29.1	33.3		33.7	34.2	34.0	

Sample size may vary in each variable due to missing data. All data adjusted for sample weights. ^a^ SES index: calculated from four single variables (mother’s education, father’s education, economic status, description of household). SES index categories based on tertile distribution. ^b^ BMI: body mass index (*n* = 1092); weight status categories assigned according to IOTF standards [27]; for girls 13–18 years old according to age-sex-specific BMI cut-offs; for girls > 18 years old according to cut-offs for girls at age 18. SD–standard deviation. *n*-number. *p*-value: significance level of Kruskal–Wallis test for mean values and chi^2^ test for percentage distribution.

**Table 3:** Dietary patterns by health and nutrition concerns: crude and adjusted logistic regression models (odds ratios with 95% confidence interval).

Outcome: Dietary Patterns	Health Concern Index			Nutrition Concern Index		
	Bottom Tertile	Middle Tertile	Upper Tertile	Bottom Tertile	Middle Tertile	Upper Tertile
‘Traditional Polish’ (ref.: bottom teritle)						
Middle tertile						
Crude model	1.46 * (1.05; 2.05)	1.00	0.94 (0.80; 1.09)	1.10 (0.78; 1.54)	1.00	1.10 (0.80; 1.52)
Adjusted model §	1.39 (0.98; 1.98)	1.00	0.93 (0.66; 1.32)	1.05 (0.74; 1.50)	1.00	1.14(0.81; 1.61)
Upper tertile						
Crude model	1.90 *** (1.37; 2.64)	1.00	0.86 (0.61; 1.21)	1.30 (0.94; 1.80)	1.00	0.91 (0.65; 1.27)
Adjusted model §	1.87 *** (1.31; 2.67)	1.00	0.84 (0.59; 1.20)	1.10 (0.78; 1.57)	1.00	0.87 (0.61; 1.24)
‘Fruit & vegetables’ (ref.: bottom teritle)						
Middle tertile						
Crude model	1.03 (0.73; 1.44)	1.00	1.15 (0.82; 1.59)	0.86 (0.62;1.19)	1.00	0.88 (0.63; 1.22)
Adjusted model §	1.13 (0.81; 1.58)	1.00	1.12 (0.79; 1.58)	1.01 (0.72; 1.41)	1.00	0.97 (0.69; 1.38)
Upper tertile						
Crude model	1.42 * (1.03; 1.97)	1.00	1.61 ** (1.15; 2.24)	0.69 * (0.49; 0.96)	1.00	1.03 (0.75; 1.42)
Adjusted model §	1.57 * (1.11; 2.22)	1.00	1.46 * (1.02; 2.10)	0.76 (0.53; 1.08)	1.00	1.01 (0.70; 1.45)
‘Fast food & sweets’ (ref.: bottom teritle)						
Middle tertile						
Crude model	1.10 (0.79; 1.54)	1.00	0.83 (0.60; 1.15)	1.04 (0.90; 1.20)	1.00	0.87 (0.63; 1.19)
Adjusted model §	1.09 (0.77; 1.53)	1.00	0.81 (0.57; 1.15)	0.94 (0.66; 1.34)	1.00	0.86 (0.61; 1.21)
Upper tertile						
Crude model	1.66 ** (1.20; 2.30)	1.00	0.96 (0.69; 1.34)	1.43 * (1.03; 1.98)	1.00	0.91 (0.65; 1.26)
Adjusted model §	1.52 * (1.08; 2.13)	1.00	1.02 (0.72; 1.44)	1.30 (0.92; 1.83)	1.00	0.97 (0.69; 1.37)
‘Dairy & fats’ (ref.: bottom teritle)						
Middle tertile						
Crude model	1.13 (0.81; 1.56)	1.00	0.68 * (0.49; 0.95)	1.06 (0.75; 1.50)	1.00	0.90 (0.65; 1.25)
Adjusted model §	1.14 (0.81; 1.62)	1.00	0.69 * (0.48; 0.98)	0.98 (0.68; 1.39)	1.00	0.94 (0.67; 1.34)
Upper tertile						
Crude model	1.63 ** (1.17; 2.26)	1.00	0.96 (0.69; 1.33)	1.02 (0.75; 1.39)	1.00	0.95 (0.69; 1.32)
Adjusted model §	1.66 ** (1.18; 2.34)	1.00	0.96 (0.68; 1.36)	0.97 (0.69; 1.37)	1.00	0.93 (0.66; 1.31)

§ Odds ratio adjusted for age (years), socioeconomic status (continuous variable measured as SES index which was calculated from four single components: mother’s education, father’s education, economic status, description of household). BMI (as categorical variable according to IOTF standards [27]; for girls 13–18 years old according to age-sex-specific BMI cut-offs; for girls > 18 years old according to cut-offs for girls at age 18 (as categorical variable). Statistically significant: * *p* < 0.05, ** *p* < 0.01, *** *p* < 0.001, **** *p* < 0.0001 (Wald’s test).

## Supplementary Materials

The following are available online at www.mdpi.com/2072-6643/9/6/613/s1, Table S1: Sample distribution by categories of perceived health and nutrition concerns; Table S2: Components of dietary patterns identified by principal component analysis (factor loadings); Table S3: Perceived health and nutrition concerns by socioeconomic status, body weight status and dietary patterns (mean (SD), in points).
