# Supplementary material for: Perceived Health and Nutrition Concerns as Predictors of Dietary Patterns among Polish Females Aged 13–21 Years (GEBaHealth Project)
Source: Nutrients. 2017 Jun 16;9(6):613. doi: 10.3390/nu9060613 (PMC5490592; doi:10.3390/nu9060613)
Supplement: Supplementary file 1 [file nutrients-09-00613-s001.zip › nutrients-199755-supplementary.pdf]

Supplementary Mterials

Table S1. Sample distribution by categories of perceived health and nutrition concerns

Category	Health Concern Index			Nutrition Concern Index		
	Range (points)	Sample size (n)	Sample percentage (%)	Range (points)	Sample size (n)	Sample percentage (%)
total sample	0–18	1107	100.0	0–42	1107	100.0
tertile distribution						
bottom tertile	0–4	355	32.1	0–12	339	30.6
middle tertile	5–8	424	38.3	13–19	417	37.6
upper tertile	9–18	328	29.6	20–42	351	31.8
a priori distribution [a]						
low concerns	0–5	420	37.9	0–13	386	34.9
neutral concerns	6–12	587	53.1	14–28	645	58.3
high concerns	13–18	100	9.0	29–42	76	6.8

All data adjusted for sample weights. [a] cut-offs were calculated as 1/3 and 2/3 of minimum-maximum range. Health Concern Index and Nutrition Concern Index were calculated as a sum of points assigned to each statements based on 7-point Likert scale starting from ‘definitely not’ (0 point) through ‘neither not nor yes’ (3 points) to ‘definitely yes’ (6 points).

Table S2. Components of dietary patterns identified by principal component analysis (factor loadings).

	Foods	Factor 1: ‘Traditional Polish’	Factor 2: ‘Fruit & vegetables’	Factor 3: ‘Fast food & sweets’	Factor 4: ‘Dairy & fats’
Food frequency consumption of [a]:	White bread (including biscuits, muffins)	0.65			
	Potatoes	0.52			
	Red meats	0.51			
	Margarine or butter	0.45			0.45
	Fried chicken	0.42			
	Wholegrain bread	-0.48			
	Green salad		0.57		
	Fruit (without juices)		0.55		
	Prepared vegetables		0.55		
	Beans		0.45		
	French fries or potato chips or corn chips or popcorn			0.71	
	Hamburgers or cheeseburgers			0.60	
	Ice cream			0.52	
	Doughnuts or pastries or cake or cookies			0.50	
	Salad dressings or mayonnaise (not diet)			0.42	
	Cheese or cheese spread				0.54
	Whole milk				0.49
Food intake variety by food groups [b]:	Meats, fish and eggs	0.60			
	Fats	0.45			0.43
	Vegetables		0.60		
	Fruit		0.54		
	Sweets and snacks			0.47	
	Cereals and potatoes				0.56
	Dairy products				0.54
Eigenvalues		4.36	2.39	1.68	1.44
Variance explained (%) [c]		14.5	9.0	5.6	4.8

Factor loadings of ≤ |0.40| are not shown in the table for simplicity. Sorted by loadings from 1st to 4th factor. All data adjusted for sample weights. [a] Food frequency consumption was expressed in points (range 0–4 points). [b] Food intake variety was expressed in foods consumed per week (with

ranges from 0–4 to 0–14 foods/week). [c] Total variance in dietary variables explained by 4 patterns is 33.9%

Table S3. Perceived health and nutrition concerns by socioeconomic status, body weight status and dietary patterns (mean (SD), in points).

Variable		Number	Health Concern Index [c]	*p*-value	Nutrition Concern Index [d]	*p*-value
Total sample		1107	6.9 (3.7)		17.1 (6.6)	
Age	13-15 y	326	6.5 (3.4)		16.8 (6.1)	
	16-18 y	367	7.2 (3.8)	0.0911	17.2 (7.1)	0.6516
	19-21 y	414	7.1 (3.8)		17.3 (6.6)	
Residence	Rural area	521	7.1 (3.6)		17.2 (6.9)	
	Town	348	6.6 (3.7)	0.0770	17.1 (6.7)	0.9975
	City	238	7.1 (3.8)		16.9 (5.9)	
SES Index [a]	Low	401	6.9 (3.8)		17.1 (6.7)	
	Medium	339	7.0 (4.0)	0.9042	17.2 (7.0)	0.9314
	High	367	6.8 (3.3)		17.0 (6.2)	
BMI category [b]	Underweight	110	4.6 (2.5)		13.8 (4.8)	
	Normal weight	849	6.8 (3.5)	<0.0001	16.9 (6.5)	0.0599
	Overweight	115	9.4 (3.9)		21.3 (7.2)	
	Obese	18	9.7 (4.6)		20.2 (6.9)	
Dietary patterns						
'Traditional Polish'	Bottom tertile	367	7.6 (3.8)		17.4 (6.8)	
	Middle tertile	364	6.8 (3.5)	<0.0001	17.4 (6.5)	0.0599
	Upper tertile	376	6.4 (3.7)		16.6 (6.6)	
'Fruit and vegetables'	Bottom tertile	364	6.7 (3.4)		16.5 (6.4)	
	Middle tertile	367	6.9 (3.5)	0.5826	17.0 (6.9)	0.0550
	Upper tertile	376	7.2 (4.1)		17.7 (6.6)	
'Fast foods and sweets'	Bottom tertile	365	7.4 (3.8)		17.3 (6.4)	
	Middle tertile	365	6.9 (3.6)	0.0009	17.2 (6.7)	0.1821
	Upper tertile	377	6.5 (3.6)		16.8 (6.8)	
'Dairy and fats'	Bottom tertile	366	7.4 (3.7)		17.4 (6.7)	
	Middle tertile	365	6.8 (3.6)	0.0005	16.9 (6.6)	0.5634
	Upper tertile	376	6.6 (3.7)		17.0 (6.6)	

SD–standard deviation. Sample size may vary in each variable due to missing data. All data adjusted for sample weights. [a] SES index: calculated from four single variables (mother's education, father's education, economic status, description of household); SES index categories based on tertile distribution. [b] BMI: body mass index (n = 1092); weight status categories assigned according to IOTF standards [27]; for girls 13–18 years old according to age-sex-specific BMI cut-offs; for girls > 18 years old according to cut-offs for girls at age 18. [c] Index range: 0-18 points. [d] Index range: 0–42 points, both indices were calculated as a sum of points assigned to each statement based on 7-point Likert scale starting from 'definitely not' (0 point) through 'neither not nor yes' (3 points) to 'definitely yes' (6 points). *p*-value: significance level of Kruskal–Wallis test.
